# A Sensitive Urinary Lipoarabinomannan Test for Tuberculosis

**DOI:** 10.1371/journal.pone.0123457

**Published:** 2015-04-23

**Authors:** Beston Hamasur, Judith Bruchfeld, Paul van Helden, Gunilla Källenius, Stefan Svenson

**Affiliations:** 1 Department of Clinical Science and Education, Karolinska Institutet, Stockholm, Sweden; 2 Unit of Infectious Diseases, Institution of Medicine, Karolinska Institutet Solna and, Karolinska University Hospital, Solna, Stockholm, Sweden; 3 DST/NRF Centre of Excellence for Biomedical TB Research, US/MRC Centre for TB Research, Division of Molecular Biology and Human Genetics, Faculty of Health Sciences, Stellenbosch University, Tygerberg, South Africa; Centre National de la Recherche Scientifique - Université de Toulouse, FRANCE

## Abstract

We have previously developed a diagnostic test for tuberculosis based on detection of mycobacterial lipoarabinomannan (LAM) in urine. The method depended on a laborious concentration step. We have now developed an easy to perform test based on a magnetic immunoassay platform, utilizing high avidity monoclonal antibodies for the detection of LAM in urine. With this method the analytical sensitivity of the assay was increased 50-100-fold compared to conventional ELISA. In a pilot study of HIV-negative patients with microbiologically verified TB (n=17) and healthy controls (n=22) the sensitivity of the test was 82% and the specificity 100%. This is in stark positive contrast to a range of studies using available commercial tests with polyclonal anti-LAM Abs where the sensitivity of the tests in HIV-negative TB patients was very low.

## Introduction

WHO reports that, if left untreated, each person with active tuberculosis (TB) infects between 10 to 15 new individuals annually. Therefore, effective control of TB requires disruption of transmission chains, which in turn requires early and accurate detection/diagnosis paired with appropriate treatment. Despite the enormous global burden of TB, present tests for diagnosis of active TB have severe limitations, and none are of the most urgently needed point-of-care (PoC) type [1].

In low income countries the most commonly used diagnostic test still is direct sputum smear microscopy, a technique that was developed in the 1880s and since then remains largely unchanged. It only detects about half of pulmonary TB patients and is particularly ineffective for diagnosis of TB in young children, in patients co-infected with HIV, and in patients with extra-pulmonary TB. Although it is often described as a simple technology, microscopy requires a high level of training and diligence and is labor intensive.

### Methods that directly/ indirectly measure the presence of tubercle bacilli

A rapid field adapted method that directly or indirectly measures the presence of live tubercle bacilli in a given host at the sampling time would be the best diagnostic tool for TB control programs. Basically such a method could aim for detecting either mycobacteria specific antigens (*i*.*e*. immunologically active components of Mtb) in the infected host (urine, blood, sputum, cerebrospinal fluid or homogenates from biopsies) or nucleic acids, or possibly Mtb specific antibodies. There have been many attempts to use determination of specific antibody titers (different ELISA, EIA formats) to diagnose TB both in man and animals. So far none of these methods have proven specific or sensitive enough to be of any diagnostic value. In fact, WHO has formally rejected the use of such current tests for humans [2].

Detection of mycobacterial antigens in urine as a diagnostic test has several potential advantages compared with all currently used diagnostics. Urine samples are simple to collect, process and store. Urine is a particularly attractive specimen in young children, who are unable to produce sputum.

The major Mtb surface antigen is lipoarabinomannan (LAM), which is the major glycolipid surface component of the Mtb cell wall and may account for up to 15% of the total bacterial weight. LAM consists of a mannan polysaccharide backbone with branched oligoarabinosyl containing saccharide side chains; the former is covalently linked to a phosphatidyl inositol lipid moiety [3–5]. During TB disease LAM in a soluble form is released both from metabolically active and degrading bacterial cells [6,7]. Hence, we assumed that in active TB disease LAM occurs in serum and subsequently may be cleared through the kidneys and occur in urine in an antigenically intact form. Furthermore, as LAM is a carbohydrate antigen, and thus inherently heat-stable, LAM may be detectable by sensitive immunological techniques, even after heat treatment of urine samples. At least in theory, the amount of LAM in the urine should reflect the bacterial load, metabolic activity and/or rate of degradation of the bacteria, and hence permit a semi-quantitative assessment of the infectious status and response to anti-bacillary treatment.

In 2001 we reported that LAM was indeed excreted in urine from mice experimentally inoculated with Mtb [8] and in patients with active TB [8] using: i) an antigen capture ELISA, and ii) a dipstick test utilizing polyclonal antibodies raised against LAM. In the antigen capture ELISA the original urine samples were boiled, desalted and concentrated 50-fold.

Subsequently field studies in Ethopia [9,10] showed that by using the antigen capture ELISA and applying the laborious concentration of urine, proof of concept was established and showed a relatively high specificity (83%) and a sensitivity of 66%, indicating that a more sensitive assay using a less complicated concentration technique should work as a PoC test.

Based on these early data, a urine LAM ELISA test was produced by Chemogen Inc. (Portland, ME, USA) and a commercial version of this test was marketed as the Clearview TB ELISA [(Alere Inc. (formerly Inverness Medical Innovations Inc.), Waltham, MA, USA]). Unfortunately this test has certain deficiencies, since polyclonal rabbit antibodies (Abs) of low avidity and specificity were used [11]. The Clearview LAM test has generated a series of clinical studies, and a systematic review and meta-analysis regarding the use of the test was published in 2011 [12]. Thus in seven studies, assessing test accuracy in microbiologically confirmed cases only, estimates of sensitivity ranged from 13% to 93% and specificity from 87% to 99%. When results were stratified by HIV status in five studies, mean sensitivity in HIV-negative patients was 14% (range 7–24%) and in HIV-positive patients 51% (32–69%). The sensitivity of the test was 3–53% higher in HIV-positive than HIV-negative subgroups and was highest with advanced immunosuppression [12]. This review concluded that the Clearview ELISA test had several characteristics that made it attractive for diagnosing active TB, but that it had suboptimal sensitivity for routine clinical use (*11*). The Clearview as well as a strip test (Alere Determine TB LAM Ag test) are however sold for TB diagnosis in HIV patients with advanced immunosuppression.

It seems clear that this type of test has good potential for PoC of TB, but that an increase in sensitivity is needed, as well as further development to make it reproducible and feasible for large-scale production, providing speed, ease-of-use, low cost and robustness.

To reduce the time required for target detection, a minimal amount of sample manipulation is essential. The sensitivity of the detection method has to be high enough to reduce/eliminate the need for target amplification and complicated enrichment steps.

To this end we produced monoclonal Abs (mAbs) directed against LAM [13], using in-house produced immunogens. This work resulted in a large series of mAbs of extremely high avidity and affinity. Clones were transferred to Genovac (now Aldevron Freiburg, http://www.aldevron.com/antibody/overview/) for large scale preparation of mAbs, and ELISA technology was transferred to Future Diagnostics (http://www.future-diagnostics.nl) to set up production of a standard antigen capture ELISA test based on these high avidity mAbs. This LAM ELISA test was evaluated by FIND in several test sites in Africa but sensitivity still proved to be inadequate for routine diagnosis (unpublished data). However, our anti-LAM mAbs were used in the development of a single-molecule sensitive fluorescence-linked immunosorbent assay as an analytical platform for the detection of LAM. This format proved to be about 3 orders of magnitude more sensitive than the comparable standard ELISA assays [14]. Samples containing as little as 600 LAM molecules per μl produced a distinct signal. This methodology, although it requires relatively sophisticated and expensive equipment, served as an additional proof of principle that the sensitivity of our diagnostic approach could be increased by making use of this anti-LAM mAb.

Based on our concept of detection of *Mtb* specific antigens in body fluids we have developed simple amplification methods in order to detect LAM in samples from both smear-positive and smear-negative TB patients. We describe a magnetic immunoassay platform, utilizing magnetic nanoparticles (MNP) in order to concentrate the antigen. With this method we have increased the analytical sensitivity of the assay 50-100-fold compared to conventional ELISA.

## Materials and Methods

### Ethics statement

The Regional Ethical Review Board at Karolinska Institutet in Stockholm approved of the study. Patients were included after giving their verbal and written informed consent when the nature and possible consequences of the study had been fully explained. Laboratory samples were coded and analysed in a blinded fashion.

### Material and Reagents

Magnetic nanoparticles (MNP) were purchased from Chemicell GmbH/Germany.

mAbs CSU-35 and CSU40 (directed at LAM) produced under the NIH, TBVTRM Contract with Colorado State University (CSU) were used as reference mAbs.

### Preparation of anti-LAM mAbs

Cell wall preparations from Mtb H37Rv were isolated and LAM was purified [15], and anti-LAM mAbs were produced as previously described [13]. The isotypes of the mAbs were identified by ELISA, using alkaline phosphatase-conjugated goat antimouse IgG subclass specific antibodies (Sigma Chemical Co, USA) and LAM as coating antigen. The mAbs of IgG1 subclass with highest affinity were chosen for further studies.

### Assessments of the titre and avidity of the anti- LAM mAbs

#### 1. ELISA

The relative titres of the mAbs were determined by ELISA. Wells of polystyrene microplates (Maxisorb, Nunc, Denmark) were coated with 100 *μ*l of purified LAM (10 *μ*g/ml) in 0.05 M carbonate buffer, pH 9.6, at room temperature overnight. The plates were washed three times with rinsing buffer (PBS containing 0.05% Tween), and then blocked with 0.5% casein for 1 h at 37°C. After washing, 100 *μ*l of serial dilutions of each mAb were added to the wells and incubated for 1 h at 37°C. After washing with rinsing buffer, 100 *μ*l of alkaline phosphatase-conjugated goat antimouse IgG (Sigma Chemical Co., diluted 1/2000 in PBS) was added to each well and the plates were incubated for a further 1 h at 37°C. After subsequent washings, the plates were developed at room temperature using p-nitrophenyl phosphate (Sigma Chemical Co.) as substrate and the colour reaction was measured by increase in absorbance at 405 nm using an ELISA reader (Dynatech, MR 5000).

#### 2. Surface Plasmon Resonance (SPR)

The interaction between mAbs and LAM was analyzed using surface plasmon resonance (SPR) (Biacore 2000; Biacore, GE Healthcare, Uppsala, Sweden) in order to identify the mAbs displaying the highest avidity.

Six mg of LAM was delipidated by alkaline treatment with 0.1 M NaOH for 1 h at 80°C. The pH of the suspension was adjusted to 6.0 with 1 M HCl and the lipid precipitate was removed by centrifugation. The delipidated LAM (dLAM) was then treated with 0.05 M periodate for 15 min at 4°C in the dark with stirring. After stopping the reaction with 100 μl of ethylene glycol the activated AM purified on a PD-10 column equilibrated with 0.1 M sodium bicarbonate buffer at pH 8.3.

After testing various linkers for immobilization of the activated AM to the gold surface, cystamine activation of the gold surface was chosen, introducing a 2- carbon atom long spacer arm, leaving free amino groups available for covalent conjugation to the activated AM.

The sensor chips were coated with different concentration of activated AM ranging from 0.1–0.5 mg AM on carbohydrate basis. After incubation over night at ambient temperature in the presence of 20 mg/ml sodium borohydride the chips were rinsed twice with PBS and the remaining sites on the chips were blocked with 5 mM mercapto ethanol for 20 min. The chips were then washed gently with PBS and air-dried for 2 h at room temperature. The chips were then blocked with 0.5% casein at room temperature for 1 h. After rinsing 100 μl of biotinylated anti-LAM antibody, 2ug/ml was added onto the surface of each chip. After 1 h incubation the chips were rinsed with PBS-Tween buffer. Next, the chips were incubated with 100 μl of avidin-HRP at 1:5000 dilution. After 1 h incubation the chips were rinsed gently with washing buffer and 100 μl of TMB substrate was loaded on the surface of the chip followed by 15 min incubation. The blue solution developed on the chip surface was then transferred into the wells of a microtitre plate and the reaction stopped with 50 μl of 16% sulphuric acid. The absorbance was recorded with an ELISA reader at 450 nm.

Avidity assessment of mAbs was performed using BIAcore 3000 at 30°C in PBS at 20 μl/min flow rate, and a total injection of 20 μl. For regeneration of the sensor chips different buffers were examined: glycine-HCl buffer at pH 1.5, 2.0, 2.5 and 3, and 3 M guanidine hydrochloride and 100 mM sodium hydroxide. The best condition for regeneration was 100 mM NaOH, which was then always used.

### Synthesis of gold (Au) coated MNP particles and Au MNP—mAb conjugate for the Uri-TB-direct test

MNP particles (10^13^) were resuspended in 5 ml toluene and the solution was heated to 85°C.

HAuCl_4_ 3H_2_O (0.05 g) and oleylamine (1.25 ml) in toluene (5 ml) were injected to the mixture while maintaining the temperature at 85°C for 1 h. The solution was cooled to RT to produce a dark purple solution. The particles (0.1 g) were dissolved in 20 ml phenyl ether and then mixed with 2 ml oleic acid (6 mM) and 2 ml oleylamine (4 mM) under N_2_ with vigorous stirring. 1,2-Hexadecanediol (2.85 g) was added to the solution and heated to 120°C under reflux for 2 h, then cooled to RT. The Fe_3_O_4_-Au NPs were precipitated in ethanol (approx 15 ml) and separated by either centrifugation or by a permanent magnet. The pellets were washed twice with ethanol and re-dispersed in 3–5 ml water. Tri-sodium citrate (0.04 g) was added and the pH of the resulting solution adjusted to 6.5. The solution was sonicated for 15 min in ultrasonic bath and the particles were collected by magnet or centrifugation and re-dispersed in water and sonicated again for another 15 min.

UV/visible spectra were obtained using a Molecular Devices Spectromax 384 spectrometer. TEM images were obtained using an FEI Tecnai G^2^ 120 kV TEM operated at 100 kV and visualized using analySIS software. MAbs were immobilized to the surface of the gold coated particles by an in house method (US Patent 6165468).

### Uri-TB-direct assay

The Au-MNP mAb particles were used in an assay to detect LAM in urine by detecting antibody-*Mtb* complex signals. Five ml urine samples were boiled for 15 min and after chilling they were placed in 10 ml tubes and 10 μl of the MNP-AB-Conjugate was added to each urine sample. The tubes were incubated for 15 min and then placed in a magnet-stand for 3 min. The supernatant was then removed and the particles were washed with 4 ml of PBS/Tween solution. This procedure was repeated once. Then 500 μl of a biotin-labelled mAb solution was added to each tube, vortexed briefly and incubated for 10 min. After washing, a Streptavidin-HRP Conjugate (500 μl) was added to each sample and incubated for another 10 mi. After washing 200 μl of SURE Blue TMB reagent was added per sample tube and incubated for 5 min in the dark. 100 μl of the coloured supernatant was placed in a well of a 96-well plate; 50μl of Stop Reagent was added and absorbance was read at 450 nm.

### Urine samples from patients and controls

At the TB clinic, Karolinska University Hospital, Stockholm, urine samples were collected from 17 HIV-negative adult patients (age 22–63 years) with culture and/or PCR confirmed TB (9 with pulmonary TB, and 8 with extrapulmonary TB). From each patient one random urine sample was collected in a 50 cc polypropylene tubes before onset of TB treatment. Samples from 22 adult TB naïve healthy individuals (age 28–60 years) were collected from hospital staff at South Hospital, Stockholm, All samples were immediately frozen at—20°C, and tested within one month.

## Results

### Binding capacity of mAbs

**Fig 1:**
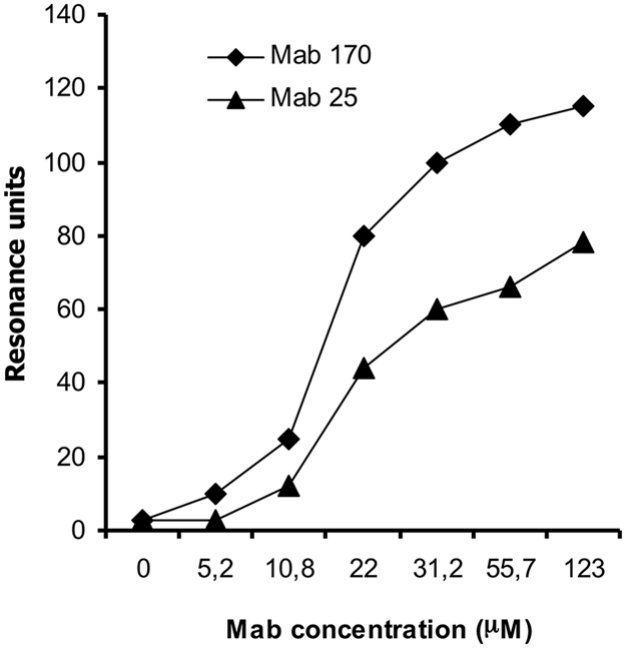
Kinetics of mAb binding to immobilized LAM at various mAb concentrations using Biacore.

**Fig 2:**
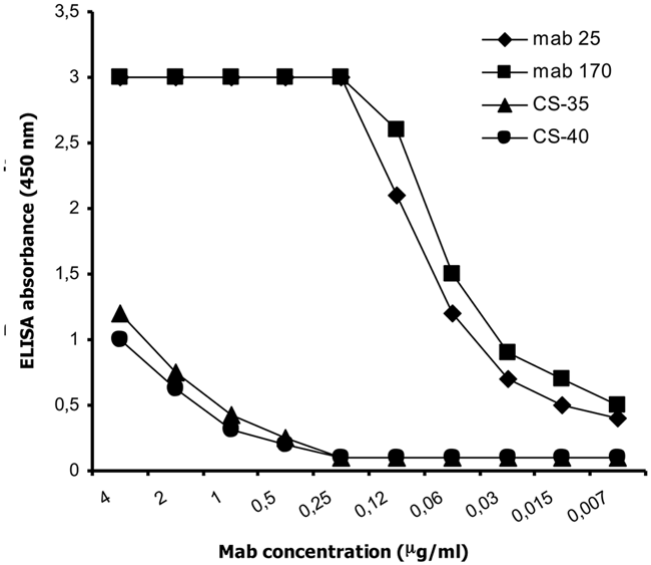
ELISA titration curve of inhouse Anti-LAM mAbs 25 and 170 compared to the reference anti-LAM mAbs CSU-35 and CSU-40.

SPR analysis revealed two mAbs out of 22 (mAb 25 and 170) with the highest avidity kD value of 3.9 x 10^-8^ and 8.0 x 10^-7^ respectively. These two mAbs also showed the best binding capacity to the LAM antigen (Fig 1). The ELISA titration curve of the same two mAbs when compared to the reference anti-LAM mAbs CSU-35 and CSU40 showed 3 logs higher binding capacity (Fig 2).

### Analytical sensitivity for detection of LAM by ELISA using mAbs

Using the high avidity mAbs 25 and 170 and urine spiked with LAM at increasing concentrations the detection limit was estimated at 1 ng/ml in ELISA (Table 1).

**Table 1:** Detection of LAM by ordinary ELISA and the Uri-TB-direct test at various concentrations of LAM (ng/ml).

LAM-spiked urine ng/ml	ELISA	Uri-TB-direct
10	2.78*	> 5.0
5	2.07	> 5.0
2.5	1.01	> 5.0
1	0.51	4.41
0.5	0.36	3.47
0.25	0.32	2.34
0.1	0.33	1.44
0.05	0.36	0.79
0	0	0.38

Results in optical density (OD) by the two different methods. The detection limit for the ELISA is ≤1 ng LAM/ml and for the **Uri-TB-direct** assay ≤ 0.05 ng LAM/ml.

* OD at absorbance 450 nm

### Analytical sensitivity of the Uri-TB-direct test format

By utilising the Au-MNP, with MAb25 as capture and MAb170 as developer mAb, the detection limit was increased 50 fold compared to the ELISA (Table 1), reaching a detection limit of 0.05 ng/ml when using a volume of 5 ml of urine. The starting amount of processed urine had an important effect on the detection limit of the Uri-TB-direct assay (Table 2). Thus the detection limit was 10 times higher when 0.5 ml urine was used as starting material, compared to when 5 ml urine was used.

**Table 2:** Effect of urine volume on detection limit of the Uri-TB-direct assay.

LAM ng/ml	0.5 ml	2 ml	5 ml
10	> 5 *	> 5	> 5
5	1.4	> 5	> 5
1	0.34	2.2	4.4
0.5	0.13	0.96	4.1
0.25	0.13	0.44	1.96
0.12	0.13	0.17	1.1
0	0.13	0.18	0.22

* OD at absorbance 450 nm

### LAM detection by Uri-TB-direct in TB patients and controls

By reading the absorbance at 450 nm a cut-off value for the sensitivity and specificity was set at an optical density (OD) of 0.35. Among urine samples from 22 healthy individuals analysed all were negative (mean OD was 0,17; range 0.12–0.30) (Table 3). Of the 17 urine samples from HIV-negative patients with culture confirmed TB 14 (82%) generated a positive result (mean OD was 0,49; range 0,21–1,36) (Table 4). Two of the 17 patients were sputum smear microscopy positive by concentration, and both these were LAM positive.

**Table 3:** Detection of LAM by the Uri-TB-direct test in urine of healthy controls.

Individual	Uri-TB-direct (OD)
1	0,16
2	0,19
3	0,2
4	0,14
5	0,2
6	0,15
7	0,18
8	0,3
9	0,18
10	0,16
11	0,18
12	0,15
13	0,12
14	0,2
15	0,2
16	0,15
17	0,14
18	0,14
19	0,16
20	0,13
21	0,21
22	0,19
Mean OD	0,17

Results in optical density (OD) at absorbance 450 n.

**Table 4:** Detection of LAM by the Uri-TB-direct test in urine of patients with microbiologically verified TB.

Patient	Pulmonary (P)/ Extrapulmonary (E)	Sputum smear microscopy*	Sputum PCR*	Mtb culture*	Uri-TB-direct (OD)
1	E (urogenital)	ND**	ND	Pos	Neg (0,21)
2	P	Pos	Pos	Pos	Pos (1,00)
3	E (abscess)	Neg	Neg	Pos	Pos (0,62)
4	P	Neg	Pos	Pos	Neg (0,27)
5	P	Pos	Pos	Pos	Pos (0,83)
6	P	Neg	Pos	Pos	Neg (0,29)
7	P	Neg	Neg	Pos	Pos (0,58)
8	E (ocular)	Neg	ND	Pos	Pos (0,43)
9	E (lymph node)	ND	ND	Pos	Pos (1,36)
10	P	Neg	Pos	Neg	Pos (1,00)
11	P	Neg	Neg	Pos	Pos (0.77)
12	E (lymph node)	ND	ND	Pos	Pos (0.78)
13	E (pleural)	Neg	Neg	Pos	Pos (0.36)
14	E (lymph node)	ND	ND	Pos	Pos (0.94)
15	P	Neg	Neg	Pos	Pos (0.37)
16	E (lymph node)	ND	ND	Pos	Pos (0,64)
17	P	ND	ND	Pos	Pos (1,14)
				Mean OD	0,49

Results in optical density (OD) at absorbance 450 n.

* Sputum samples were liquefied and decontaminated using N-acetyl-L-cysteine and NaOH (final concentration 1.5%). Portions of the decontaminated pellet were used for microscopy, culture and PCR. Smear microscopy was performed using auramine staining. For culture the material was inoculated into a Mycobacteria Growth Indicator Tube (MGIT) (Becton Dickinson, USA) and solid Lowenstein Jensen medium and incubated for up to 42 days. The Cobas TaqMan MTB test (Roche Diagnostics, Basel, Switzerland) was used for PCR.

** Not done

## Discussion

We have developed a new format of a LAM detection test, using mAbs with extremely high avidity, and increased the signal by using new nanotechnology techniques. The format of the Uri-TB-direct platform makes the test easy to perform. It includes a few pipetting and washing steps. A technician can handle 24–48 samples in about 2 h. In this study an ELISA reader was used in order to do a quantitative measurement of the LAM concentration, however, for field purposes, the ELISA reader can be replaced by either: 1) a battery operated portable ELISA reader (cost about 500 USD), or ii) by reading the colour change by the naked eye. In that case, no electricity is needed.

The amount of excreted LAM has been reported to vary between 1 ng/ml (i.e. the detection limit of the tests used) and several hundred ng/ml [16–18], depending on the clinical manifestations. Quantitative LAM detection results increase with bacterial burden [19]. Thus, smear positive patients have been shown to be more often positive in LAM ELISA than smear negative patients [17,20,21], and a positive correlation between bacterial density in sputum and urine concentration of LAM has been demonstrated [17]. The sensitivity of urine LAM testing increases with progressive HIV immune suppression (as reflected by falling CD4 cell counts), probably correlating with an increased total mycobacterial burden [18], and the concentration of LAM in urine is associated with poor prognosis in patients with HIV-associated TB [22].

Since the LAM concentration in urine of non-HIV co-infected TB patients is extremely low, in many cases in the range of picogram per ml, the “ordinary” LAM test (ELISA or strip test), which detects LAM in the range of nanogram per ml, is not sensitive enough for diagnosis in unselected TB suspects. In this study we demonstrate how one may achieve a 50-100-fold increase in urinary LAM detection using our Uri-TB-direct assay compared to previous methods.

In our previous reports using a sandwich ELISA with polyclonal antibodies, the detection limit of LAM in spiked samples was 1 ng/ml [8]. By using a laborious concentration step the analytical sensitivity of the test was increased to 20 pg/ml of urine [8,10]. In those studies the samples were boiled and then centrifuged after which the supernatant was further purified through column chromatography and the eluent was concentrated 50-fold by a rotavapor. With this concentration procedure the sensitivity of the test was estimated to be 81% in sputum smear positive Ethiopian TB patients [10]. However, this laborious method is not suited for the development of an easy to use POC test. The detection limit of our earlier sandwich ELISA using polyclonal Abs [8] without the concentration step was in the same range as the detection limit reported for a similar sandwich ELISA using polyclonal antibodies later produced by Chemogen [17], and further on by the Clearview ELISA, in the range of 1 ng/ml. In the studies utilizing the commercial tests no attempt to concentrate the urine was made, except for one study, where concentration of the urine by centrifugation through a 10 K molecular filter yielded a modest increase in sensitivity (from 33 to 38%) [18].

In our small pilot study of HIV-negative patients with verified TB and controls the specificity of our test was 100% and the sensitivity 82%. Our results are in stark positive contrast to a range of studies using the commercial tests with polyclonal anti-LAM Abs where the sensitivity of the tests in HIV-negative TB patients was very low (mean 14%) and are better than what is found in HIV-positive patients (mean 51%) [12]. In a next step the Uri-TB-direct assay in a simplified format will be evaluated in cohorts from different settings in high and low endemic areas and in various patient populations.

### In memoriam

Stefan Svenson, who was the initiator of this project, passed away unexpectedly before this manuscript could be published. We dedicate this paper to his memory.
